# CT‐based analysis of dose homogeneity in total body irradiation using lateral beam

**DOI:** 10.1120/jacmp.v5i4.1980

**Published:** 2004-11-24

**Authors:** Susanta K. Hui, R.K. Das, Bruce Thomadsen, Douglas Henderson

**Affiliations:** ^1^ Departments of Human Oncology University of Wisconsin‐Madison 600 Highland Avenue Madison Wisconsin 53792; ^2^ Departments of Medical Physics University of Wisconsin Madison Wisconsin; ^3^ Tomotherapy Incorporated 1240 Deming Way Madison Wisconsin 53717 U.S.A.; ^4^ Present address: Department of Therapeutic Radiology‐Radiation Oncology University of Minnesota 420 Delaware Street SE, Mayo Mail Code 494 Minneapolis MN 55455 U.S.A.

**Keywords:** TBI, lateral technique, lung pneumonitis, dose verification

## Abstract

A computed tomography (CT) based treatment planning system for total body irradiation (TBI) is presented and compared with the commonly practiced lateral treatment delivery. The TBI regimen has been proved to be an essential conditional regimen for patients undergoing bone marrow transplantation. The advantage of the TBI regimen with bone marrow transplantation (BMT) in hematological malignancies can be offset by toxicities arising from TBI in posttransplant complications. With the increasing survival rates, the evaluation of long‐term side effects and quality of life has become an important area of research interest. There have been several treatment techniques developed over the decades designed to achieve accurate dose delivery and dose homogeneity. This paper reports on the verification of the dose delivery for a basic lateral technique using thermoluminescent dosimeters (TLDs) placed in an anthropomorphic phantom and its correlation with CT‐based treatment planning. CT‐based treatment plans on several patients were used to evaluate the doses delivered to the whole body and critical organs. A large variation in doses delivered to the whole body was demonstrated, with some parts of the bone marrow failing to receive the prescribed dose and some critical organs, such as the lungs, receiving excessive doses. Placing the arms at the sides only partially compensates for the increased transmission of the lungs because the arms only shadow part of the lung. This study shows that CT‐based treatment planning for TBI provides precise and accurate dose calculations and allows for the correlation of clinical outcomes with the doses actually delivered to various organs.

PACS numbers: 87.53.Dq, 87.66.Xa, 87.66.Sq

## I. INTRODUCTION

The total body irradiation (TBI) treatment has been accepted as an important radiotherapy treatment for hematological malignancies and is used in conjunction with chemotherapy as a conditioning regimen for bone marrow transplantation (BMT) or peripheral blood stem cell transplantation.^(^ ^1^ ^,^ ^2^ ^)^ Total body irradiation serves two purposes: first, it provides immunosup‐pression, allowing subsequent engraftment of the transplanted stem cells; second, it contributes to eradicating a modest number of radiosensitive tumor cells, clearing the host marrow to allow repopulation with donor marrow cells. Various TBI schedules have been proposed to optimize these objectives. With increasing survival rates,^(^ ^3^ ^–^ ^6^ ^)^ evaluation of long‐term side effects and quality of life has become an important area of research interest. Total body irradiation dose, fractionation,^(^ ^7^ ^–^ ^11^ ^)^ and dose rate^(^ ^12^ ^,^ ^13^ ^)^ are primarily limited by the toxicity to the normal tissues such as lung, liver, and kidney.

Among others, pulmonary complications, renal toxicities, cataracts, and reduced pituitary function are the main long‐term affects of TBI. The short‐term side effects include mucositis, dysphasia, diarrhea, parotitis, erythema, and veno‐occlusive disease. Lung pneumonitis is the primary dose‐limiting factor. It is difficult to assess the limiting lung dose, due particularly to inaccuracies in the largely inhomogeneous dose distribution within the lungs resulting from the single‐point dose calculation model generally used.^(^ ^14^ ^,^ ^15^ ^)^ Dose‐volume histograms (DVHs) are rarely available for patients undergoing TBI, impeding further analysis of limiting pulmonary tolerance. Aldo et al.^(^ ^16^ ^)^ showed a relation between mean lung dose and lethal pulmonary complications (LPCs). Lethal pulmonary complication was observed to be only 3.8% for a patient who received inhomogeneity corrected lung dosage of up to 9.4 Gy, whereas a 19.2% LPC risk (p=0.05) was observed beyond 9.4 Gy. Morgan et al.^(^ ^9^ ^)^ have reported a 20% LPC rate for allogeneic transplanted patients using higher dose rates (>0.1Gy/min). This is consistent with the expected protective effect of lung toxicities by lowering the dose rate, based on a short component for the repair of sublethal cellular damage.

Other nonradiation variables could influence lung complications. Tait et al.^(^ ^17^ ^)^ reported an influence of graft versus host disease in the occurrence of lung complication due to infectious pneumonitis and cytomegalovirus. Treatment optimization is necessary to overcome this situation. Chemotherapy^(^ ^18^ ^)^ may also be responsible for lung toxicity.

Many trials were undertaken to understand the pretransplant and posttransplant factors causing complications for long‐term morbidity and mortality.^(^ ^19^ ^–^ ^24^ ^)^ Total body irradiation‐related complications (mainly toxicities) were studied by varying prescribed dose, dose rate, single fraction, and dose fractionation. Dose fractionation and a low–dose‐rate scheme favor reducing toxicities to the critical organs with late‐responding characteristics (lowα/β) and controlling leukomic cells of high α/β ratio and a low capacity for cellular repair processes.^(^ ^25^ ^)^ However, low‐dose, fractionated TBI appears to be significantly less effective and is associated with lower overall survival rates compared with higher doses of radiation.^(^ ^7^ ^,^ ^26^ ^)^ Although increasing the TBI dose may improve the relapse‐free survival rate, the number of toxic deaths increased.^(^ ^27^ ^)^ Over the past decade, the routine TBI dose to the target has increased to 12 Gy to – 14 Gy. Dose inhomogeneities are mainly due to tissue inhomogeneities, the contour of the human body, and beam energy. Tissue inhomogeneities cause the most pronounced dose increase for lung tissue. Compensation is sometimes made through the introduction of partially shielding lung blocks. Energy selection is based on the need to achieve higher whole‐body dose homogeneity and lower‐dose deposition to the lung (both require higher energy) and the need to avoid low doses near the surface in the buildup region (favoring low energy). A 10‐MeV photon beam is often chosen in this situation. The use of a long source‐to‐surface distance (SSD, 300 cm) serves two purposes: first, it allows a single field to cover the whole body; second, when combined with lowering the pulse repetitive rate, it reduces the dose rate to below 10 cGy/min to prevent higher dose rate‐related toxicities. In general, two parallel opposed beams are used in either anterior‐posterior (AP‐PA) or opposed lateral techniques. The AP‐PA technique requires a suitable lung block that sufficiently reduces the dose to the lung while allowing an adequate chest wall dose. Electron field boosts to the shielded chest wall may be required. The opposed lateral beam technique uses the positioning of the patient's arm as compensators for the lung.

Computed tomography (CT)‐based treatment planning can provide a detailed calculation of dose delivery to the lung for the treatment setup. Accurate anatomical dose distribution and DVHs may help in understanding what level of dose inhomogeneity is clinically significant. Better knowledge of dose tolerances could assist in further optimization of the TBI regimen, improving prevention of radiation‐induced disease. Dose inhomogeneity can lead to failure of TBI through either insufficient dose to the marrow stem cells or excessive dose to critical organs. An AAPM report^(^ ^28^ ^)^ suggested that future clinical evaluations should address uncertainties in dose delivery to the target and the normal tissue. The present study is an assessment of the dose distribution in the lungs for opposed lateral TBI fields, using CT‐based treatment.

## II. METHODS

A partial whole body Rando phantom (head through midfemur) was used for dose verification and treatment‐planning verification. A prescription dose of 13.2 Gy (1.65 Gy per fraction) was delivered using two lateral 10‐MeV fields at 300‐cm SSD. With the collimator rotated 45° and field size set to 40×40cm, this technique allows coverage of the entire body. Dose verification used LiF (lithium fluoride) thermoluminescent dosimeters (TLD – TLD 100, powdered, 5% dose uncertainty) in capsules. The TLDs were placed inside the Rando phantom at organ sites of interest, such as manubrium, lungs, xyphoid, iliac crest, and hip, for dose verification during a TBI treatment. The TLDs were calibrated and the linearity with respect to dose verified for a range from 50 cGy to 500 cGy.

Computed tomography was performed on the rando phantom using 5‐mm thick slices on a GE Light Speed CT scanner. During the imaging, both arms were kept resting at the side, simulating the lateral treatment, in which arms act as a natural compensator for the lung. A Pinnacle™ (Philips, Milpitas, CA) radiation treatment‐planning workstation was used for dose calculation. Dose distributions (dose/fraction) were normalized to a point located at the level of the hip in the middle of the body.

Three patients of various sizes also were imaged and planned in the same manner as the phantom. Each patient's study contained more than 200 total Dicom images. Dose‐volume histograms were created for the target and individual sensitive organs. Dose as a function of area was studied on CT images taken at three different levels (upper, middle, and lower) in the lung for all three patients to investigate the average contribution of arm shielding of the lung and the dose variation due to the shape of the lung and arm. At each of the three levels of lung, a DVH was generated, and the area covered by the arm was estimated.

## III. RESULTS

### A. TLD measurement in phantom

**Figure 1:**
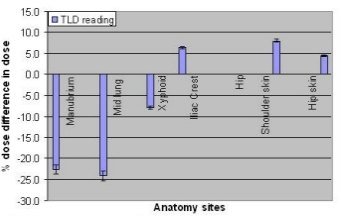
Doses measured with TLD compared with prescribed doses at the selected points in the Rando phantom irradiated with parallel‐opposed lateral beams. The error bars indicate the 5% uncertainty in TLD measurements.

TLD‐based dose verification for the Rando phantom shows significant dose variation compared with the intended dose delivery. This is shown in Fig. 1 as the dose difference in various anatomical sites as a percentage of the prescribed dose.

**Figure 2:**
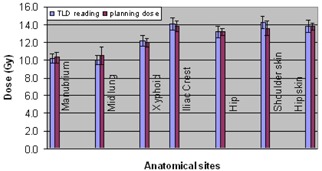
Doses in phantom measured with TLD and calculation based on CT treatment plan.

The CT‐based treatment‐plan dose is compared with TLD measurement in Fig. 2. Results of both methods agree well within the limit of uncertainty of dose verification methods.

### B. Patient simulations

**Figure 3:**
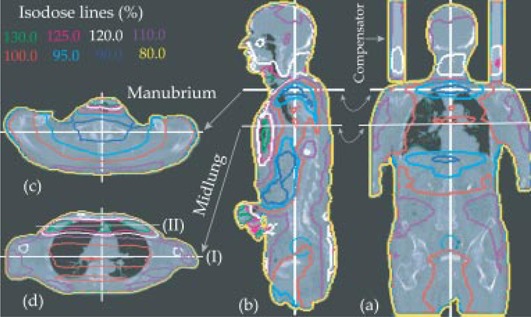
Isodose distribution shown on a patient CT, as calculated by treatment‐planning system for lateral‐opposed beam. The horizontal white lines show the levels of the transverse images (manubrium and midlung) to the left of the sagittal images. (a) coronal; (b) sagittal; (c) transverse manubrium; (d) transverse midlung image.

**Figure 4:**
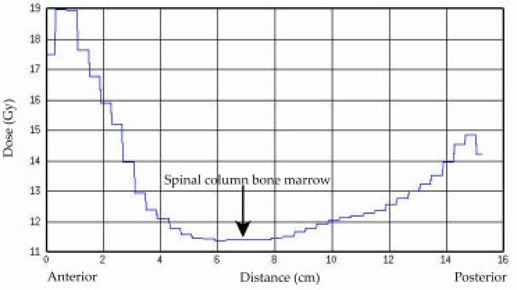
Midline dose profile in the anterior‐posterior direction at the level of the manubrium.

The dose variation in one of the patients can be seen in Fig. 3, particularly in the transverse images through the lungs at the level of the manubrium and midlung in (Fig. 3c) and (Fig. 3d), respectively. White lines in each image plane indicate the positions for the other orthogonal planes. (Figure 3a) and (Fig. 3b) represent coronal and sagittal views, respectively, for the same patient. Part of the bone marrow blocked by the shoulder at the manubrium region received a dose less than 80% of that prescribed, as shown in the dose profile in Fig. 4.

**Figure 5:**
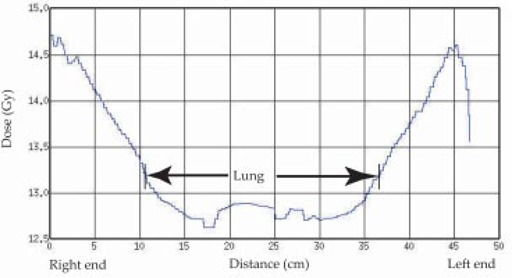
Midline dose profile in the lateral direction at the transverse level of the midlung, along line I in (Fig. 3d).

**Figure 6:**
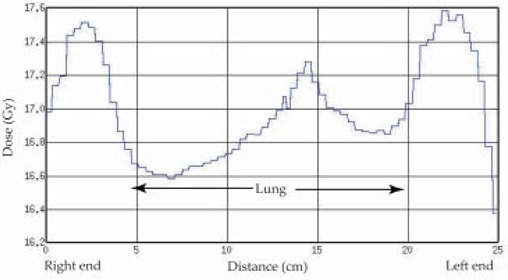
Midline dose profile in the lateral direction at the transverse level of the midlung, along line II in (Fig. 3d).

The maximum dose variation was found in the lung. Two lateral dose profiles were created at the level of the midlung: Fig. 5 at the middle of the transverse image at the level I in (Fig. 3d), and Fig. 6 at 5 cm from the anterior edge of the transverse image at level II in (Fig. 3d). A dose minimum of around 12.5 Gy is observed in Fig. 5, whereas a maximum of 17.6 Gy is observed in Fig. 6. The fraction of the partial volume of the lung that receives doses higher than that prescribed varies with patient size and arm position. Inferior to the manubrium, some part of the lung received doses 32% above that prescribed.

The dose variations in umbilical and pelvic sites were mostly within 10%. At the same time, a wrist placed on the umbilicus received a dose 30% higher than that prescribed. The eyes also showed higher doses (14.75 Gy) than planned, even when using a 5‐cm acrylic compensator $\left(\text{density}\;1.185g/{\text{cm}}^{3}\right)$ for the head.

A quantitative analysis of dose variations, averaged over measured data for three patients, in various anatomical sites is presented in Table 1.

**Table 1:** Quantitative analysis of dose variations for three patients in various anatomic sites from CT‐based treatment planning calculations

	Average dose (Gy) (% SD)	
Anatomy sites	Minimum	Maximum
mediastinal/shoulder	11.13 (4.49)	18.19 (2.91)
upper lung	11.80 (4.92)	12.87 (1.01)
midlung	12.03 (7.5)	16.93 (3.13)
lower lung	12.83 (4.29)	17.48 (2.8)
zyphoid	11.3 (2.65)	15.29 (6.67)
umbilical	13.23 (5.44)	17.56 (4.78)
hip	13.17 (1.14)	16.21 (3.39)
femur	12.33 (6.33)	17.07 (6.27)

The result of the lung dose‐area study is shown in Table 2 at the three levels, along with the corresponding shoulder/arm area. The area covered by the shadow of the arm decreases inferiorly, whereas the area of the lung peaks near the middle slices. There is a trend for higher doses to higher fractions of the lung area toward the lower lung.

**Table 2:** Lung dose‐area study at three levels of lung is tabulated for different dose coverage

			% area exposed inside lung				
Anatomy sites	Lung area (cm^2^)	Shoulder shadow area (cm^2^)	>13Gy	>14Gy	>15Gy	>16Gy	>17Gy
upper lung	8.62	15.23	21.41	0.00	0.00	0.00	0.00
midlung	21.55	10.80	80.81	46.22	36.80	22.06	2.21
lower lung	18.81	8.22	90.98	66.54	49.33	24.00	0.71

## IV. DISCUSSION

Some of the bone marrow at the level of the manubrium region was underdosed by more than 20%. This is clearly due to the placement of the arms at the side, which protects the lung but also reduces the dose at this level. The point‐dose measurement at the middle of the lung (midsternum) confirmed the dose reduction inside the lung to almost 75% of the prescribed dose. The doses to the other anatomical sites were within 5% to 10% of that prescribed. Additional bolus or compensation would be necessary to achieve better dose homogeneity. The calculated dose distribution agrees well with dose measured using TLDs as shown in Fig. 2. Underdose to the bone marrow can have the immediate effect of rejecting the donor marrow and increasing the relapse rate in the long term. Bone marrow in other parts of the body received a dose equal to or greater than the prescribed dose. Uncertainty included in the calculated dose to the TLDs was due to the uncertainty (±2mm) in determination of the exact location of the TLDs in the CT image. It is not understood what level of dose uncertainty in bone marrow could affect the clinical result.

Throughout the body, the maximum dose variation was found in the lung due to the shape of the lung, density changes, and the variations in arm shape adjacent to the lung. With lateral fields, the arms are placed at the side of a patient to compensate for lack of attenuation in the lung. However, part of the lung, not covered by the arms, gets very high doses. This phenomenon is demonstrated in the CT image (Fig. 3) as well as in the dose profile showing a higher dose in the anterior portion of the lung (Fig. 6) compared with the midlung (Fig. 5). The lateral lung dose was under 12.5 Gy under the shadow of the arm, while high doses (16.6 Gy to 17.6 Gy) were observed in the anterior part of the lung due to the absence of the arm shadow and the low density of the lung itself.

**Figure 7:**
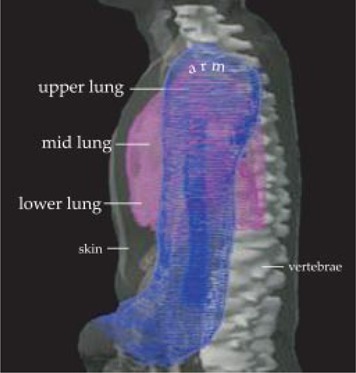
Beam's‐eye view of one of the lateral fields. Body skin is a gray transparent color, the lung is in the pink wire frame, and the arm is in the blue wire frame.

As shown in Table 2, the lung slice area increases inferior direction to the level of the heart where the left lung space is minimal. The arm width and thickness decrease inferiorly. Consequently, the midlung and lower lung received a relatively high dose. For visual clarification, a 3D lateral projection (beam's‐eye view) of the exposed lung is shown in Fig. 7. The lung is partially blocked by the arm. Although the upper lung was blocked fully by the arm, the increase in the dimensions of the lung inferiorly and simultaneous decrease in arm size (thickness in both lateral and anterior‐superior), resulting in significant uncompensated lung volume exposure. Of course, the variation in the size of the lung and in the arm and the day‐to‐day variation in placement of the arm during treatment could lead to more variations in dose distribution. The effect of this high lung dose on lung toxicities and on survival rate could be inferred from future clinical trials.

The acrylic compensator reduces dose to the head, but the eyes still receive a dose of about 14.5 Gy. Blocking the eyes could reduce that dose, but the block would also reduce the dose to the bone marrow of the orbit. The other anatomy sites were mostly within 10% of the prescribed dose.

## V. CONCLUSIONS

Our study showed that positioning the arms along the sides of the patient partially compensated for the low density of the lung. However, in the volume of the lungs not covered by the arms, the radiation exposure increased with increasing lung volume, resulting in a dose 20% to 32% higher than that prescribed. High‐dose regions in the lung are of concern because the whole body prescription dose was determined to reduce lung‐related toxicities. The problem of highdose exposure to the lungs needs to be addressed in future clinical treatments in order to further reduce lung toxicity. Additional compensation may protect the lung but may also underdose the bone marrow at the manubrium and ribs, leading to increased chance of relapse. Similar studies are needed for the AP‐PA technique. The CT‐based treatment is able to account for dose inhomogeneity and provides anatomy‐based dose distribution, such as bone marrow and sensitive organs. Such information would be particularly useful for clinical outcome studies correlated with dose. With proper dosimetric control, the radiobiological aspects of TBI could be studied in a quantitative manner.

## ACKNOWLEDGMENTS

We thank Bhudatt Paliwal for his support for this project.

## References

[1] Gilson D , Taylor RE . Total body Irradiation. Br J Radiol. 1997;70:1201–1203. 9505836 10.1259/bjr.70.840.9505836

[2] Thomas ED , Storb R , Clift RA , et al. Bone marrow transplantation. N Engl J Med. 1975; 292:832–838. 234595 10.1056/NEJM197504172921605

[3] Roncadin M , Arcicasa M , Bortolus R , et al. Feasibility of total body irradiation in chronic lymphatic leukemia and low‐grade non‐Hodgkin's lymphomas. Cancer Invest. 1991;9(4):403–407. 1884246 10.3109/07357909109084637

[4] Blume KG , Kopecky KJ , Henslee‐Downey JP , et al. A prospective randomized comparison of total body irradiation‐etoposide versus busulfan‐cyclophosphamide as preparatory regimens for bone marrow transplantation in patients with leukemia who were not in first remission: A Southwest Oncology Group Study. Blood 1993;81: 2187–2193. 8471778

[5] McGlave PB , Arthur DC , Kim TH , et al. Successful allogeneic bone marrow transplantation for patients in the accelerated phase of chronic granulocytic leukemia. Lancet 1982;2:625–627. 6125774 10.1016/s0140-6736(82)92737-4

[6] Barett A . Total body irradiation (TBI) before bone marrow transplantation in leukemia: A co‐operative study from the European Group for Bone Marrow Transplantation. Br J Radiol. 1982;55:562–567. 7052186 10.1259/0007-1285-55-656-562

[7] Sabine B , Claudine H , Bernard C , Raymond M . Total body irradiation before allogeneic bone marrow transplantation: Is more dose better? Int J Radiat Oncol Biol Phys. 2001;4:1071–1077. 10.1016/s0360-3016(00)01491-7 11240249

[8] Socie G , Devergie A , Girinsky T , et al. Influence of the fractionation of total body irradiation on complication and relapse rate for chronic myelogenous leukaemia. Int J Radiat Oncol Biol Phys. 1991;20:397–404. 1995523 10.1016/0360-3016(91)90048-9

[9] Morgan T , Falk P , Kogut N , et al. A comparison of single dose and fractionated total‐body irradiation on the development of pneumonitis following bone marrow transplantation. Int J Radiat Oncol Biol Phys. 1996;36:61–66. 8823259 10.1016/s0360-3016(96)00246-5

[10] O'Donoghue JA . Fractionated versus low dose‐rate total body irradiation: Radiobiological consideration in the selection of regimes. Radiother Oncol. 1986;77:241–247. 10.1016/s0167-8140(86)80035-4 3544085

[11] Cosset JM , Socie G , Dubray B , et al. Single dose versus fractionated total body irradiation before bone marrow transplantation: Radiobiological and clinical consideration. Int J Radiat Oncol Biol Phys. 1994;30:477–492. 7928476 10.1016/0360-3016(94)90031-0

[12] Scarpati D , Frassoni F , Vitale V , et al. Total body irradiation in acute myeloid leukemia relapse. Int J Radiat Oncol Biol Phys. 1989;17:547–552. 2674077 10.1016/0360-3016(89)90105-3

[13] Kim TH , Rybka W , Lehnert S , et al. Interstitial pneumonitis following total body irradiation for bone marrow transplantation using two different dose rates. Int J Radiat Oncol Biol Phys. 1985;11:1285–1291. 3891697 10.1016/0360-3016(85)90243-3

[14] Briot E , Dutreix A , Bridier A . Dosimetry for total body irradiation. Radiother Oncol 1990;18(Suppl):241–247. 10.1016/0167-8140(90)90175-v 2123356

[15] Planskoy B , Tapper PD , Davis FM . Physical aspects of total body irradiation at the Middlesex Hospital (UCL group of hospitals), London 1988‐1993. In‐vivo planning and dosimetry. Phys Med Biol. 1996;41:2327–2343. 8938029 10.1088/0031-9155/41/11/006

[16] Aldo D , Andres J , Maria F , et al. Lethal pulmonary complications significantly correlate with individual assessed mean lung dose in patients with hematologic malignancies treated with total body irradiation. Int J Radiat Oncol Biol Phys. 2002;52:483–488. 11872296 10.1016/s0360-3016(01)02589-5

[17] Tait RC , Burnet AK , Robertson AG , et al. Subclinical pulmonary function defects following autologous and allogeneic bone marrow transplantation: Relationship to total body irradiation and graft versus‐host‐disease. Int J Radiat Oncol Biol Phys. 1991;20:1219–1227. 2045296 10.1016/0360-3016(91)90231-r

[18] Down JD , Berman AJ , Warhol M , et al. Late tissue‐specific toxicity of total body irradiation and busulfan in a murine bone marrow transplant model. Int J Radiat Oncol Biol Phys. 1989;17:109–116. 2663795 10.1016/0360-3016(89)90377-5

[19] Clift RA , Buckner CD , Thomas ED , et al. The treatment of acute non‐lymphoblastic leukemia by allogeneic marrow transplantation. Bone Marrow Transplant 1987;2:243–258. 3332174

[20] Tallman MS , Kopecky KJ , Amos D , et al. Analysis of prognostic factors for the outcome of marrow transplantation or further chemotherapy for patients with acute nonlymphocytic leukemia in first remission. J Clin Oncol. 1989;7:326–337. 2645386 10.1200/JCO.1989.7.3.326

[21] Hill GR , Krenger W , Ferrara JL . The role of cytokines in acute graft‐versus‐host disease. Cytokines Cell Mol Ther. 1997;3:257–266. 9740354

[22] Sanders JE . Bone marrow transplantation for pediatric leukemia. Pediatr Ann. 1991;20:671–676. 1766698 10.3928/0090-4481-19911201-06

[23] Winston DJ , Ho WG , Champlin RE . Current approaches to management of infections in bone marrow transplants. Eur J Cancer Clin Oncol. 1989;5(Suppl2):S25–S35. 2693107

[24] Sanders JE , Pritchard S , Mahoney P , et al. Growth and development following marrow transplantation for leukemia. Blood 1986;68:1129–1135. 3533180

[25] Wheldon TE . The radiological basis of total body irradiation. Br J Radiol. 1997;70:1204–1207 and Steel GG , Wheldon TE . The radiation biology of paediatric tumours. Pinkerton R, Plowman N, editors. Paediatric oncology. London: John Wiley; 1991. 9505837 10.1259/bjr.70.840.9505837

[26] Corvò R , Lamparelli T , Bruno B , et al. Low‐dose fractionated total body irradiation (TBI) adversely affects prognosis of patients with leukemia receiving an HLA‐matched allogeneic bone marrow transplant from an unrelated donor (UD‐BMT). Bone Marrow Transplant. 2002;30:717–723. 12439693 10.1038/sj.bmt.1703701

[27] Clift RA , Buckner CD , Appelbaum FR , et al. Allogeneic marrow transplantation in patients with chronic myeloid leukemia in the chronic phase: A randomized trial of two irradiation regimens. Blood 1991;77:1660–1665. 2015394

[28] American Association of Physics in Medicine (AAPM) Report No 17: The physical aspects of total and half body photon irradiation; 1986.

